# Neural correlates of cigarette health warning avoidance among smokers

**DOI:** 10.1016/j.drugalcdep.2016.01.025

**Published:** 2016-04-01

**Authors:** George Stothart, Olivia Maynard, Rosie Lavis, Marcus Munafò

**Affiliations:** a School of Experimental Psychology, University of Bristol, UK; b MRC Integrative Epidemiology Unit (IEU) at the University of Bristol, UK; c UK Centre for Tobacco and Alcohol Studies, UK

**Keywords:** Tobacco, Smoking, Health warnings, vMMN, LPP

## Abstract

**Background:**

Eye-tracking technology has indicated that daily smokers actively avoid pictorial cigarette package health warnings. Avoidance may be due to a pre-cognitive perceptual bias or a higher order cognitive bias, such as reduced emotional processing. Using electroencephalography (EEG), this study aimed to identify the temporal point at which smokers’ responses to health warnings begin to differ.

**Method:**

Non-smokers (*n* = 20) and daily smokers (*n* = 20) viewed pictorial cigarette package health warnings and neutral control stimuli. These elicited Event Related Potentials reflecting early perceptual processing (visual P1), pre-attentive change detection (visual Mismatch Negativity), selective attentional orientation (P3) and a measure of emotional processing, the Late Positive Potential (LPP).

**Results:**

There was no evidence for a difference in P1 responses between smokers and non-smokers. There was no difference in vMMN and P3 amplitude but some evidence for a delay in vMMN latency amongst smokers. There was strong evidence for delayed and reduced LPP to health warning stimuli amongst smokers compared to non-smokers.

**Conclusion:**

We find no evidence for an early perceptual bias in smokers’ visual perception of health warnings but strong evidence that smokers are less sensitive to the emotional content of cigarette health warnings. Future health warning development should focus on increasing the emotional salience of pictorial health warning content amongst smokers.

## Highlights

• Smokers implicitly and explicitly avoid cigarette health warnings.
• Smokers and non-smokers lectroencephalography (EEG) responses to cigarette health warnings were compared.
• Smokers show no difference in early perceptual processing (P1, vMMN and P3).
• Smokers show reduced later cognitive responses (Late Positive Potential).
• Smokers show reduced sensitivity to cigarette health warnings emotional content

## 1 Introduction

Cigarette package health warnings increase awareness of the health risks of smoking, and attention to health warnings has been shown to lead to meaningful changes in behaviour, such as forgoing cigarettes and contemplating quitting smoking (Hammond, 2011). However, interviews with regular smokers have found that 36% reported making some attempt at avoiding the warnings, such as hiding them, using a cigarette case, or requesting a specific package to avoid a particular warning (Hammond et al., 2004). Using eye-tracking technology, we have previously measured health warning avoidance at a more implicit level and found that daily smokers also actively avoid pictorial health warnings by directing visual attention away from them within the first seconds of viewing (Maynard et al., 2014).

Recent neuroimaging research has highlighted some of the possible neural mechanisms involved in the processing of health warnings amongst smokers. Smokers show less activation in, and connectivity between, the medial pre-frontal cortices and the insula when viewing aversive smoking related stimuli compared to aversive non-smoking related stimuli, i.e., they are less responsive to drug-specific aversive stimuli (Dinh-Williams et al., 2014a, Dinh-Williams et al., 2014b). The level of brain activity whilst viewing aversive smoking related images has also been correlated with intentions to quit and reductions in subsequent smoking behaviour (Wang et al., 2013). Interestingly these changes occur in the context of an increase in brain activity amongst substance users when presented with substance related images (see Littel et al., 2012 for a meta-analysis and review), suggestive of a specific adaptation of neural responses to health warnings that dissociates them from other smoking related cues.

What remains unclear from the current neuroimaging research is the cognitive locus of this reduction in neural activity, i.e., where in the perceptual/attentional/cognitive processing stream does the consequence of being a smoker impact on the processing of health warnings? One possible explanation is that through repeated exposure to warnings, a pre-cognitive perceptual bias may develop, leading to the reduced attentional salience of the warnings. Previous research has shown early perceptual biases *towards* smoking-related stimuli among smokers (Versace et al., 2011). It is therefore possible that a stimulus presenting an anti-smoking message (e.g., a health warning) may be associated with an early perceptual bias *away* from it. Alternatively (or additionally), avoidance behaviours may be a result of higher order cognitive biases, such as reduced emotional processing, that functions to inhibit afferent perceptual information (Loeber et al., 2011).

Using electroencephalography (EEG), the aim of this study was to identify the temporal point at which smokers’ responses begin to differ and consequently identify the underlying biases. Four Event Related Potentials (ERPs) were used to address this question, the visual P1, visual Mismatch Negativity (vMMN), the P3 and the Late Positive Potential (LPP). The amplitude and latency of these components reflect the integrity and efficiency of the early stages of perception, change detection and attentional orientation through to higher level cognitive emotional processing. The visual P1 response is believed to represent the processing of stimulus characteristics and visuo-spatial selection (Clark et al., 1994, Clark and Hillyard, 1996, Di Russo et al., 2002, Proverbio et al., 2007). It is typically unaffected by complex visual features and often overlooked in favour of later components, however Versace et al. (2011) demonstrated cue-reactivity like increases in P1 amplitude to cigarette stimuli amongst smokers. vMMN provides a measure of pre-attentive visual change detection and discriminative processing of stimuli (see Kimura et al., 2011, Stefanics et al., 2015 for reviews). It is sensitive to changes in simple physical characteristics (e.g., Tales et al., 1999) as well as more complex characteristics of visual images, such as emotional content during face processing (Kecskés-Kovács et al., 2013) and symmetry (Kecskés-Kovács et al., 2012). It is elicited in response to a rare deviant stimulus embedded amongst repeating standard stimuli. Importantly for the current study, the extent to which the deviant oddball stimulus differs from preceding standard stimuli affects the magnitude of the vMMN response. Therefore if the deviant stimulus is of high valence to one group (e.g., smokers) they will show a larger vMMN response compared to a second group (e.g., non-smokers) for whom the deviant stimulus is of a lower valence.

The P3 component provides the first index of selective attentional orientation and is proposed to represent the updating of working memory representations of incoming stimuli (Polich et al., 2008). The Late Positive Potential (LPP) provides a measure of higher order cognitive biases and reflects the cortical prioritisation of emotional information during visual processing (Brown et al., 2012, Littel and Franken, 2011). It is typically elicited in passive viewing paradigms, manifests as a midline centroparietal ERP 300–400 ms following stimulus onset, and is larger following the presentation of both pleasant and unpleasant compared to neutral visual stimuli (see Hajcak et al., 2011 for a review).

Two separate EEG paradigms were designed to elicit these specified ERP components. An ‘oddball’ perceptual paradigm was designed to elicit the P1, vMMN and P3. A separate passive viewing paradigm was designed to elicit a higher order cognitive LPP response, avoiding any contamination of the LPP response with preceding task-associated responses, e.g., P3.

We hypothesised that daily smokers would show reduced neural responses to pictorial health warnings, in particular familiar health warnings. The point in the processing stream at which smokers and non-smokers begin to differ will elucidate the underlying cause of any changes. Long-term habituation of the perceptual/attentional responses would be predicted to manifest as reduced amplitudes/delayed latencies of the earlier ERP components (e.g., P1, vMMN, P3). By contrast, a reduced cognitive emotional response would be predicted to manifest as a reduction in the amplitude/delay in the latency of the LPP.

## 2 Materials and methods

### 2.1 Study design

This was an EEG study of visual perception of pictorial health warning labels, with a between-subjects design, using two separate paradigms: an oddball paradigm and a passive viewing paradigm. Testing took place at the University of Bristol (ethics approval code: 12121). The study was conducted according to the revised Declaration of Helsinki 2013 and Good Clinical Practice guidelines, and the study protocol was registered on the Open Science Framework prior to commencing testing (https://osf.io/zea2t/).

### 2.2 Participants

Daily smokers (*n* = 20) were defined as smoking at least 5 cigarettes a day, smoking their first cigarette of the day within one hour of waking. Non-smokers (*n* = 20) were defined as not having smoked more than 100 cigarettes in their lifetime. Smokers’ CO reading was required to be higher than 3 parts per million (ppm). Participants were recruited from the general population, were aged between 18 and 40 years, had English as their first language, and had normal or corrected to normal vision and hearing.

### 2.3 Materials

Participants completed the Edinburgh Handedness Inventory (EHI; Oldfield, 1971), the Fagerström Test for Nicotine Dependence (FTND; Heatherton et al., 1991), the Questionnaire of Smoking Urges (QSU-brief; Cox et al., 2001) and the Quitting Smoking Contemplation Ladder (QSCL; Biener and Abrams, 1991).

The oddball paradigm used cigarette pack stimuli, comprised of cigarette branding on the top 40% of the pack and either a cigarette pack health warning (*n* = 20), or a control image of an object (*n* = 20), or a landscape (*n* = 20) on the bottom 60% of the pack. Branded cigarette pack images were 10 popular UK tobacco brands (bought in February 2014). Control object and landscape images were sourced from the International Affective Picture System (IAPS; Lang et al., 2008) database. Images were selected based on the following criteria: neutral emotional valence ratings (objects *M* = 5.03, SD = 0.79; landscapes *M* = 4.99, SD = 0.48), low arousal ratings (objects *M* = 2.60, SD = 0.53; landscapes *M* = 3.63, SD = 0.74), and not containing images of people or faces.^1^ All IAPS images had a black border added to match the health warning images. In order to explore the impact of health warning familiarity on EEG responses, health warnings comprised 10 pictorial health warnings taken from the 11 European Union (EU) pictorial warnings currently used in the UK and therefore familiar to smokers (hereafter ‘UK warnings’), and 10 EU warnings not used in the UK and therefore unfamiliar to smokers (hereafter ‘non-UK warnings’), please see Supplementary material for images of all the health warnings and cigarette packs used. UK and non-UK warnings were matched for health warning effectiveness based on pre-study piloting, (four questions assessing health warning effectiveness) with 40 participants (20 smokers, 20 non-smokers).

**Fig. 1:**
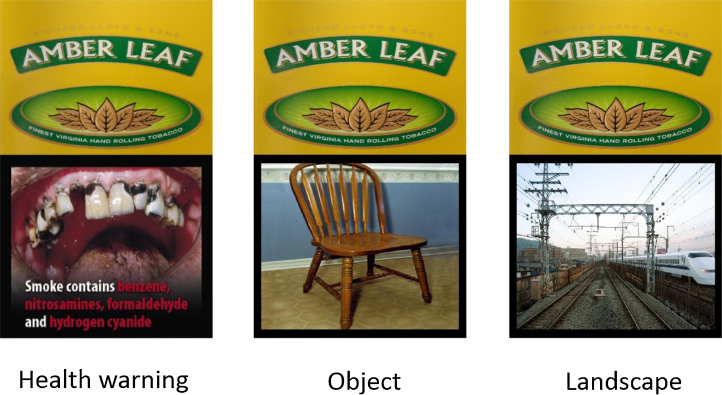
Example of the three stimulus types in the oddball paradigm.

Each branded pack image was combined with each of the 20 health warnings as well as 20 control object images and 20 landscape images, creating a total of 600 stimuli, see Fig. 1 for examples of the stimuli. Cigarette pack images were presented on screen in their actual size (5 cm × 8.5 cm). The passive viewing paradigm consisted of the 20 health warning stimuli and 20 control images used in the oddball paradigm, presented centrally on screen, (8 cm × 10 cm), without the cigarette pack.

### 2.4 Procedure

Upon arrival between 10 a.m and 4 p.m, an expired breath carbon monoxide (CO) measurement was taken to confirm smoking status. Participants then completed the EHI and smokers also completed the FTND and the QSU-brief. Participants sat 55 cm from the computer screen and completed the oddball paradigm, followed by the passive viewing paradigm.

During the oddball paradigm, participants completed three blocks, in which they were shown 800 ‘standard’, 100 ‘deviant’ and 100 ‘target’ stimuli in total. Stimuli were randomly selected for each trial from the block of 200 stimuli containing the appropriate combination of cigarette pack and health warning or control image. Their task was to respond to all target stimuli by pressing a hand-held button in their right hand. Throughout the task, participants were instructed to attend to an audio-book story, presented through headphones. Participants were told that they would need to answer questions on the content of the story at the end of the study (this was not actually the case) to ensure that visual stimuli were passively, rather than actively attended to, an important pre-requisite for the elicitation of vMMN. Stimuli were presented for 200 ms, with a randomised inter-stimulus interval of 500–700 ms. Table 1 shows the three blocks in which health warning stimuli appeared once as a standard, deviant and a target. This allowed for difference waveforms to be calculated based on the subtraction of the neural response to *physically identical stimuli* that differed solely in their status as standard, deviant or target. This also helped to control for the presence of text in the health warning stimuli, but not in the control stimuli. The order of blocks was counterbalanced across the participant sample and stimuli conditions were presented pseudo-randomly, such that deviant and target stimuli were always preceded by at least two standard stimuli. This task lasted for approximately 30 min.

During the passive viewing paradigm, participants received instructions to attend to the stimuli presented to them on screen. Each of the health warning (*n* *=* 20) and control object stimuli (*n* *=* 20) were presented five times, totalling 200 image presentations in a single block lasting approximately 12 min. Images were randomly presented for 1500 ms each, with a randomised inter-stimulus interval of 1500–3000 ms. Stimuli were presented using Presentation software v.12.2 (Neurobehavioral Systems, Inc).

Participants then completed subjective 9-point Likert scale ratings of each of the 20 health warning stimuli. Each health warning was presented and participants were asked: ‘How effective is this image?’ and ‘How familiar is this image?’ in a counterbalanced order. Smokers then completed the QSU-brief and the QSCL. Participants were then debriefed, given the opportunity to ask questions, asked for their final consent and reimbursed £12.

#### 2.4.1 EEG recording

EEG signals were sampled at 1000 Hz from 32 Ag/AgCl electrodes fitted on a standard electrode layout elasticised cap using a BrainAmp DC amplifier (Brain Products GmbH) with a common FCz reference and online low-pass filtered at 250 Hz. Impedances were below 5 kΩ. Recordings were analysed offline using BESA software v5.3 (BESA GmbH). Artifacts including blinks and eye movements were corrected using BESA automatic artifact correction (Berg and Scherg, 1994) and any remaining epochs containing artifacts > ± 100 μV were rejected. The rejection rate never exceeded 10% of trials for each participant and stimulus. Epochs of −100 to 600 ms for the oddball paradigm and −100 to 1500 ms for the passive viewing paradigm were defined around stimulus onset and baseline corrected using the pre-stimulus interval (−100 to 0 ms). A 0.01 Hz high pass and a 40 Hz low-pass filter were applied.

#### 2.4.2 ERP analysis

**Fig. 2:**
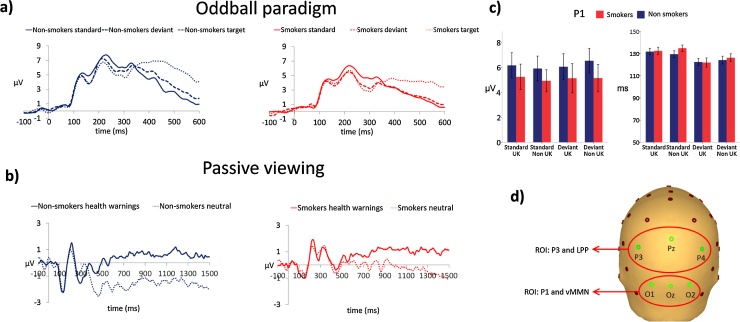
(a) Grand average waveforms for non-smokers and daily smokers for the oddball paradigm. Responses to health warnings when presented as standards, deviants and targets measured at the occipital region of interest (average of electrodes O1, Oz, O2). (b) Grand average waveforms for non-smokers and daily smokers for the oddball paradigm. Responses to health warnings and control stimuli, measured at the parietal region of interest (average of electrodes P3, Pz, P4). (c) P1 peak amplitudes and latencies in response to standard and deviant stimuli, error bars indicate the standard error of the mean. (d) Region of Interest (ROI) electrode groupings for P1, vMMN, P3 and LPP analysis.

Bespoke electrode regions of interest (see Fig. 2d) and reference channels were chosen for the oddball paradigm and passive viewing analyses, in order to optimise the measurement of each component. P1, vMMN and P3 were measured in response to the oddball paradigm, LPP was measured in response to the passive viewing paradigm. P1 and vMMN are typically measured at occipital electrode sites, therefore the values of three occipital electrodes O1,Oz,O2 were averaged to form an occipital region of interest and data were re-referenced to a common average reference based on previous analysis protocols (e.g., Stothart et al., 2015, Stothart and Kazanina, 2013). P3 and LPP are typically measured at parietal electrode sites therefore the values of three parietal electrodes P3,Pz,P4 were averaged to form a parietal region of interest (see Fig. 2d) and data were re-referenced to a virtual linked mastoid based on the optimal analysis protocol described in Hajcak et al. (2011). Averaging across electrodes that show consistent and comparable activity has been shown to be more reliable than using single electrodes (Huffmeijer et al., 2014).

P1 peak amplitude and latency was measured as the maximum positive value between 80–150 ms post stimulus onset and was calculated for responses to both health warnings and control stimuli, when presented as standard and deviant stimuli.

vMMN, P3 and LPP were all analysed using difference waveforms, i.e., by subtracting an individual’s neural response to one condition from another. This technique helped to control for inter-individual variability in ERP magnitudes that arise from variations in individuals’ cortical anatomy.

To calculate the vMMN, the averaged response to stimuli when presented as standards were subtracted from the averaged response to the *same stimuli* when presented as deviants to create a difference waveform. vMMN peak amplitude and latency was measured as the largest negative peak deflection in the difference waveform during the epoch 130–340 ms.

To calculate the P3, the averaged response to stimuli when presented as targets were subtracted from the averaged response to the *same stimuli* when presented as standards to create a difference waveform. P3 peak amplitude and latency were calculated as the largest positive peak deflection in the difference waveform during the epoch 300–600 ms.

To calculate the passive viewing paradigm LPP, the averaged response to health warnings was subtracted from the averaged response to control images. In order to establish the LPP onset times and durations in a data-driven manner rather than choosing arbitrary time windows, sequential one sample *t*-tests were applied to the difference waveforms using sequential one sample *t*-tests applied to the difference waveforms for each group (Guthrie and Buchwald, 1991). The strength of this approach is that it allows for the empirical rather than subjective identification of onset and duration of any statistically meaningful differences between two signals. The consecutive time points necessary to indicate an epoch of difference between the responses were obtained from a simulation using an autocorrelation estimated from the data. Intervals with values of *p* < 0.05 that lasted for the required duration (71 consecutive time points, i.e., 71 ms, for the non-smokers, and 35 for the smokers) were accepted as different epochs.

### 2.5 Statistical analysis

P1 was examined in a 2 (smoking status: smokers vs non-smokers) × 2 (region of use: UK vs non-UK) × 2 (condition: standard vs deviant) ANOVA. vMMN and P3 responses to health warning stimuli were examined separately in 2 (smoking status: smokers vs non-smokers) × 2 (region of use: UK vs non-UK) ANOVA. vMMN and P3 responses to control stimuli were examined in a one-way (smokers vs non-smokers) ANOVA. Sequential *t*-tests identified clear temporal differences between the two groups in the onset of the LPP. In order to examine the interaction with region of use, mean LPP amplitudes were calculated separately for the early (373–833 ms) and the late (834–1500 ms) epochs and examined in a 2 (smoking status: smokers vs non-smokers) × 2 (region of use: UK vs non-UK) ANOVA. Data were analysed using IBM SPSS Statistics for Windows, v.21.0.

The number of correct responses and the mean response time to targets in the oddball paradigm were examined in a one-way ANOVA (smoking status: smokers vs non-smokers). Subjective familiarity and effectiveness of the health warnings were examined in a 2 (smoking status: smokers vs non-smokers) × 2 (region of use: UK vs non-UK) ANOVA.

## 3 Results

### 3.1 Characteristics of participants

Participant characteristics are shown in Table 2. One-way ANOVA indicated that participants did not differ with regards to age (*F*_(1,38)_ = 0.77, *p* = 0.385) but, as expected, daily smokers had higher levels of expired carbon monoxide than non-smokers (*F*_(1,38)_ = 37.0, *p* *<* 0.001). Fisher’s Exact Test indicated that there was no difference in the number of males and females in the two groups (*p = *0.341).

### 3.2 Behavioural data

One-way ANOVA indicated that there was no difference (*F*_(1,38)_ = 0.84, *p* = 0.364) in the mean number of target stimuli identified in the oddball task among non-smokers 95.4% (SD11.3) and smokers 97.9% (SD3.9). There was also no difference (*F*_(1,38)_ = 0.81, *p* = 0.373) in mean reaction time to target stimuli for non-smokers (495 ms SD55) and smokers (512 ms SD60) for smokers. These data also indicate high levels of compliance with the task among both smokers and non-smokers.

Repeated measures ANOVA demonstrated that participants in this study rated the UK health warnings as being more effective than the non-UK health warnings (*F*_(1,38)_ = 33.9, *p* < 0.001) despite pre-study piloting suggesting that there was no difference in effectiveness between these health warnings. Smokers rated the health warnings as less effective than non-smokers (*F*_(1,38)_ = 7.87, *p* = 0.008), but there was no evidence of a smoking status by region of use (UK vs non-UK) interaction (*F*_(1,38)_ = 0.00, *p* = 0.962). UK health warnings were rated as more familiar than non-UK warnings (*F*_(1,38)_ = 30.9, *p* < 0.001) but there was no main effect of smoking status on familiarity ratings (*F*_(1,38)_ = 0.02, *p* = 0.898). A smoking status by region of use interaction was also observed (*F*_(1,38)_ = 8.10, *p* = 0.007). Bonferroni corrected post-hoc tests indicated that both non-smokers and smokers rated the UK health warnings as more familiar than the EU health warnings, but this effect was larger among smokers (*t*_(1,19)_ = 5.0, *p* < 0.001) than non-smokers (*t*_(1,19)_ = 2.4, *p* = 0.014).

### 3.3 EEG analyses

**Fig. 3:**
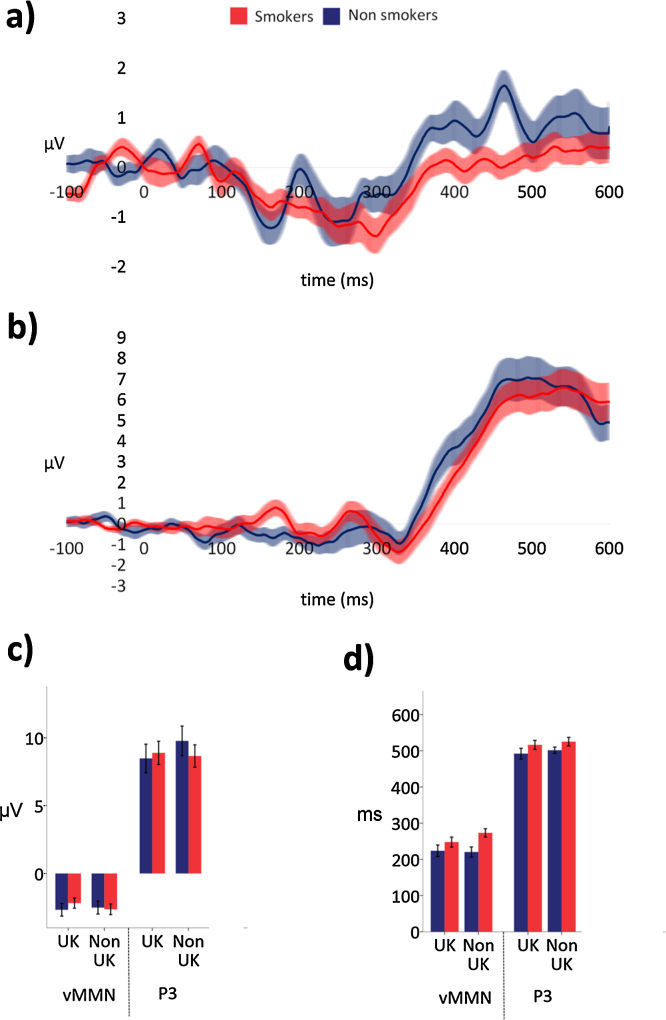
a) vMMN difference waveforms (i.e., responses to health warnings as deviants subtracted from responses to health warnings as standards) measured at the occipital region of interest (average of electrodes O1, Oz, O2), illustrating the vMMN response for non-smokers and daily smokers, shaded areas indicate the standard error of the mean. (b) P3 difference waveforms (i.e., responses to health warnings as targets subtracted from responses to health warnings as standards) measured at the parietal region of interest (average of electrodes P3, Pz, P4) illustrating the P3 response for non-smokers and daily smokers, shaded areas indicate the standard error of the mean. (c) vMMN and P3 peak amplitudes for UK and Non-UK health warnings, error bars indicate the standard error of the mean. (d) vMMN and P3 peak latencies for UK and Non-UK health warnings, error bars indicate the standard error of the mean.

**Fig. 4:**
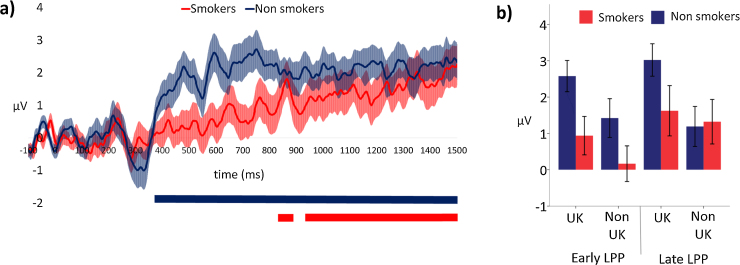
(a) Difference waveforms (i.e., responses to health warnings subtracted from responses to control stimuli) illustrating the LPP for non-smokers and daily smokers. Shaded areas indicated the standard error of the mean. Coloured horizontal bars indicate the epochs of significant difference (p< 0.05) between responses to health warning and control stimuli for each group. (b) Early and late LPP mean amplitudes for non-smokers and daily smokers for UK and non-UK health warnings. Error bars indicate the standard error of the mean.

Grand average waveforms are presented in Fig. 2, and difference waveforms for the oddball paradigm and passive viewing paradigm presented in Fig. 3, Fig. 4.

#### 3.3.1 Perceptual and attentional processing—oddball paradigm

##### P1

There was no evidence of main effects of smoking status (*F*_(1,38)_ = 0.54, *p* = 0.467) region of use (*F*_(1,38)_ = 0.25, *p* = 0.874), or condition (*F*_(1,38)_ = 0.61, *p* = 0.441) on P1 amplitudes. There was no evidence of an interaction between smoking status and condition (*F*_(1,38)_ = 0.27, *p* = 0.607) or smoking status and region of use (*F*_(1,38)_ = 1.25, *p* = 0.271). There was some evidence for an interaction between condition and region of use (*F*_(1,38)_ = 5.99, *p* = 0.019), with P1 responses to standards greater than deviants in response to UK stimuli, and smaller than deviants in response to non-UK stimuli (see Fig. 2c).

There was no evidence of an effect of smoking status (*F*_(1,38)_ = 0.27, *p* = 0.607) or region of use (*F*_(1,38)_ = 0.75, *p* = 0.392) on P1 latencies. There was clear evidence for an effect of condition (*F*_(1,38)_ = 17.6, *p* < 0.001) on P1 latencies such that responses to standards were slower than to deviants (Standards: *M* = 132 ms, SD = 13; Deviants: *M* = 123 ms,SD = 13). There was no evidence for any interactions between smoking status, region of use or condition.

##### vMMN

A clear vMMN in response to health warning stimuli when presented as deviants was observed in both groups (see Fig. 3a). A 2 (smoking status: smokers vs non-smokers) × 2 (region of use: UK vs non-UK) ANOVA revealed no evidence of an effect of smoking status (*F*_(1,38)_ = 0.10, *p* = 0.755) or region of use (*F*_(1,38)_ = 0.63, *p* = 0.431) on vMMN amplitude, and no interaction (*F*_(1,38)_ = 2.80, *p* = 0.102).

There was evidence for an effect of smoking status on vMMN latency (*F*_(1,38)_ = 5.09, *p* = 0.030) with smokers showing a delay in their vMMN response (*M* = 261 ms, SD = 57) compared to non-smokers (*M* = 221 ms, SD = 67). There was no evidence for an effect of region on vMMN latency (*F*_(1,38)_ = 1.24, *p* = 0.272), and no interaction between smoking status and region (*F*_(1,38)_ = 2.16, *p* = 0.150).

No clear vMMN was observed in either group in response to control stimuli. This was unexpected and possibly due to the relatively low arousal content of the control stimuli in comparison to the health warnings. Importantly, a one-way ANOVA (smoking status: smokers vs non-smokers) revealed no effect of smoking status on the mean amplitude of the difference waveform during this period (*F*_(1,38)_ = 1.02, *p* = 0.320), i.e., although the absence of vMMN to control stimuli was unexpected it was consistent across the two groups.

##### P3

A clear P3 in response to health warning stimuli when presented as targets was observed in both groups (see Fig. 3b). A 2 (smoking status: smokers vs non-smokers) × 2 (region of use: UK vs non-UK) ANOVA revealed no evidence of an effect of smoking status (*F*_(1,38)_ = 0.07, *p* = 0.791) or region of use (*F*_(1,38)_ = 2.13, *p* = 0.153) on P3 amplitude. There was an interaction between the effect of smoking status and region of use on P3 amplitude (*F*_(1,38)_ = 4.43, *p* = 0.042) reflecting non-smokers' increased P3 response to non-UK health warnings, (see Fig. 3c).

A 2 (smoking status: smokers vs non-smokers) × 2 (region of use: UK vs non-UK) ANOVA on P3 latency revealed no evidence of an effect of smoking status (*F*_(1,38)_ = 2.97, *p* = 0.093), region of use (*F*_(1,38)_ = 0.81, *p* = 0.375) or an interaction between smoking status and region (*F*_(1,38)_ = 0.001, *p* = 0.975) (see Fig. 3d).

A one-way ANOVA (smoking status: smokers vs non-smokers) revealed no effect of smoking status on P3 amplitude to control stimuli when presented as targets (*F*_(1,38)_ = 0.07, *p* = 0.796).

##### Perceptual and attentional processing summary

There was no evidence for an effect of smoking status on the amplitude of early perceptual (P1), change detection (vMMN) or attentional orientation (P3) responses. There was no effect of smoking status on the latency of the early perceptual (P1) response, however there was evidence that change detection (vMMN) latency was delayed in smokers.

#### 3.3.2 Cognitive emotional processing—passive viewing paradigm

Sequential t-tests identified a clear LPP in both groups, but with considerable differences in onset time (see Fig. 4a). LPP onset was 373 ms for non-smokers and 833 ms for smokers and both continued to the end of the measurement epoch (1500 ms). During the early LPP epoch, (i.e., 373–833 ms), a 2 (smoking status: smokers vs non-smokers) × 2 (region of use: UK vs non-UK) ANOVA revealed a larger LPP amongst non-smokers than smokers (*F*_(1,38)_ = 5.16, *p* = 0.029). Fig. 2 shows that the difference between the groups was driven by a stronger response to health warning stimuli amongst non-smokers, while responses to neutral stimuli were comparable to smokers. UK health warnings elicited a larger LPP than non-UK warnings (*F*_(1,38)_ = 10.42, *p* = 0.003), and there was no evidence of an interaction (*F*_(1,38)_ = 0.41, *p* = 0.524).

During the later LPP epoch, (i.e., 834–1500 ms), there was no clear evidence of a difference in LPP amplitude between smokers and non-smokers (*F*_(1,38)_ = 8.09, *p* = 0.374) although UK health warnings again elicited a larger LPP than non-UK warnings (*F*_(1,38)_ = 6.20, *p* = 0.017). There was a trend towards a smoking status by region interaction (*F*_(1,38)_ = 3.19, *p* = 0.082), driven by the large LPP among non-smokers when viewing UK health warnings (see Fig. 4b).

## 4 Discussion

Our study reveals large differences between smokers and non-smokers in higher level cognitive emotional processing, preceded by small delays in the attentional orientation towards health warnings. Specifically, we found no evidence for an early perceptual bias (P1) or explicit attentional orientation to health warnings among smokers. Pre-attentive change detection (vMMN) was delayed but not reduced in magnitude amongst smokers. It is unlikely that these differences can be explained by increased familiarity with the warnings among smokers, as similar results were observed for both familiar (UK) and unfamiliar (EU) health warnings. The greatest differences were observed in higher level cognitive emotional processing. Specifically, the LPP response was significantly delayed and reduced in amplitude amongst smokers. Overall smokers showed a delayed attentional orientation responses and a delayed and reduced later emotional response, although statistically the evidence is modest and would benefit from replication. These findings lend support to our previous finding that daily smokers avoid health warnings (Maynard et al., 2014).

In addition, we found that the LPP response was greater when both non-smokers and smokers view warnings used in the UK as compared with those that are not. The LPP is known to be sensitive to the emotional intensity of a stimulus, and does not habituate with repeated presentations (Codispoti et al., 2006). We therefore speculate that the LPP difference between UK and non-UK health warnings was more likely due to the increased effectiveness of the UK health warnings than the increased familiarity. Together, these data indicate that in order to design effective tobacco health warnings, rather than focusing on adapting the low-level perceptual characteristics of the health warnings such as location, contrast, and size, the focus should instead be upon changing the content of the health warnings, in order to increase their emotional salience. Specifically, the effects on attention of changes to the framing of health warnings, such as whether they are loss- or gain-framed (Toll et al., 2008), symbolic or depicting real-life scenes, and whether the messages they convey are personally relevant to smokers and realistic, should be explored. This research is therefore important for the development of evidence-based tobacco health warnings. In addition, our finding that daily smokers show a bias *away* from health warnings should be considered alongside the established literature which shows that daily smokers also display a bias *towards* cigarette packs and smoking related stimuli (Carter and Tiffany, 1999, Hogarth et al., 2003, Littel and Franken, 2007), i.e., cue reactivity. Future research should explore how these two potentially competing biases operate when cigarette packs with health warnings are presented.

There are some limitations with our study design. First, the passive viewing paradigm did not include an active control; it is therefore possible that any differences between non-smokers and smokers in cognitive emotional processing may be a result of general differences in processing of emotional stimuli. Second, health warning stimuli contained lexical and semantic information in the forming of text health warnings that was not present in the control stimuli. It is therefore not possible to distinguish whether it was the pictorial or text content that elicited the differing emotional responses between non-smokers and smokers. Future work could address these issues by introducing an active control in which non-smoking related emotional stimuli are presented and adding non-smoking related text to control stimuli. Third, the order of paradigm presentation was not counter-balanced, as the passive viewing paradigm had the potential to introduce a familiarity/habituation bias to the subsequent oddball paradigm. Fourth, the sample sizes were determined on the basis of previous work in substance use P300 literature, e.g., Littel et al. (2012). The P1 and vMMN can show high individual variability compared to the P300 and therefore larger group sample sizes may be beneficial for examining such components. Fifth, smokers’ CO readings were required to be higher than 3 parts per million (ppm), which is relatively low to guarantee smoking status. Future work should examine the neural response to individual health warnings in order to guide the development of more effective health warnings. Finally, we did not examine the difference between the impact of a smoker’s habitual tobacco brand compared to other brands on EEG responses. Future research should examine whether neural responses to health warnings and avoidance behaviour is affected by the presence of a smoker’s habitual brand.

To our knowledge, this is the first EEG study to experimentally investigate the mechanisms underlying avoidance of health warnings among daily smokers. Our findings support previous studies which have used subjective questionnaires to investigate health warning avoidance among daily smokers (Borland et al., 2011, Hammond et al., 2007, Hammond et al., 2004). Crucially EEG has helped to identify the temporal origin of the avoidance behaviour previously observed in daily smokers, and will help to direct the development of health warnings and increase their future impact.

## Conflict of interest

No conflict declared.

## Funding

This research was supported by the Medical Research Council Integrative Epidemiology Unit (MC_UU_12013/6), which is funded by the Medical Research Council and the University of Bristol.

## Contributors

GS, OMM and MRM were responsible for the study concept and design. GS programmed the experiment. RL was responsible for participant recruitment and testing. GS, RL and OMM analysed the data and all authors assisted with the interpretation of the finding. GS and OMM drafted the manuscript and all authors provided critical revision of the manuscript for important intellectual content and approved the final version for publication.

## Figures and Tables

**Table 1:** Allocation of stimuli types across the three blocks. O = Control object L = Control landscape. Difference waveforms were calculated by subtracting the averaged response to stimuli when presented as standards from the response the *same stimuli* when presented as deviants. E.g., to calculate a participant’s vMMN difference waveform for health warning stimuli the averaged response to health warnings as standards in Block 1 was subtracted from the response to health warnings as deviants in Block 2.

	Standard	Deviant	Target
Block 1	Health warning	Control (O)	Control (L)
Block 2	Control (O)	Health warning	Control (L)
Block 3	Control (O)	Control (L)	Health warning

**Table 2:** Participant characteristics.

	Non-smokers (*n* *=* 20)	Daily smokers (*n* *=* 20)	*P*-value
Sex (male)	7 (35%)	11 (55%)	0.341
Age	22.6 (3.6)	22.6 (8.3)	0.980
Expired carbon monoxide^a^	2.5 (0.8)	11.4 (6.1)	<0.001
Quitting contemplation ladder	n/a	5.4 (2.1)	n/a
FTND	n/a	3.5 (1.7)	n/a
QSU brief (pre-testing)	n/a	53.7 (17.3)	n/a
QSU brief (post-testing)	n/a	45.4 (15.5)	n/a
Cigarettes smoked per day	n/a	9.6 (4.2)	n/a
Mean effectiveness rating of UK health warning^b^	6.9 (1.0)	5.6 (1.9)	0.008
Mean effectiveness rating of non-UK health warning^b^	5.8 (1.6)	4.5 (1.8)	0.019
Mean familiarity rating of UK health warning^b^	7.1 (1.8)	7.8 (1.2)	0.186
Mean familiarity rating of non-UK health warning^b^	6.6 (2.1)	6.1 (2.1)	0.451

Values represent number (percentage) for categorical variables, and mean (standard deviation) for continuous variables.

FTND: Fagerström Test for Nicotine Dependence.

QSU: Questionnaire of Smoking Urges.

a One non-smoker did not provide a carbon monoxide reading due to a technical error.

b Effectiveness and familiarity scale 1 = not at all familiar/effective 9 = extremely familiar/effective.

## Footnotes

1 Image numbers objects: 5510, 6150, 7000, 7002, 7003, 7004, 7009, 7010, 7012, 7017, 7020, 7034, 7056, 7077, 7170, 7185, 7187, 7233, 7235, 7950. Image numbers landscapes: 5390, 5395, 5471, 5661, 5731, 5900, 6930, 7033, 7036, 7037, 7242, 7491, 7495, 7500, 7546, 7547, 7560, 7590, 7595, 7620.
