# Supplementary material for: Neural correlates of cigarette health warning avoidance among smokers
Source: Drug Alcohol Depend. 2016 Apr 1;161:155–62. doi: 10.1016/j.drugalcdep.2016.01.025 (PMC4803020; doi:10.1016/j.drugalcdep.2016.01.025)
Supplement: Supplementary file 1 [file mmc1.docx]

Supplementary Material for the Article

Neural correlates of cigarette health warning avoidance among smokers.

George Stothart^1^, Olivia Maynard^1,2,3^, Rosie Lavis^1^, Marcus Munafò^1,2,3^

^1^ School of Experimental Psychology, University of Bristol

^2^ MRC Integrative Epidemiology Unit (IEU) at the University of Bristol

^3^ UK Centre for Tobacco and Alcohol Studies

Corresponding author:

George Stothart^1^

School of Experimental Psychology,

University of Bristol,

12a Priory Road,

Bristol,

BS8 1TU,

UK.

[George.Stothart@bristol.ac.uk](mailto:George.Stothart@bristol.ac.uk)

Tel: + (44) 117 331 7894

Fax: + (44) 117 928 8588

This material supplements, but does not replace, the peer-reviewed article in

*Drug and Alcohol Dependence*.
