# Supplementary figures and images for: Neural correlates of cigarette health warning avoidance among smokers
Source: Drug Alcohol Depend. 2016 Apr 1;161:155–62. doi: 10.1016/j.drugalcdep.2016.01.025 (PMC4803020; doi:10.1016/j.drugalcdep.2016.01.025)

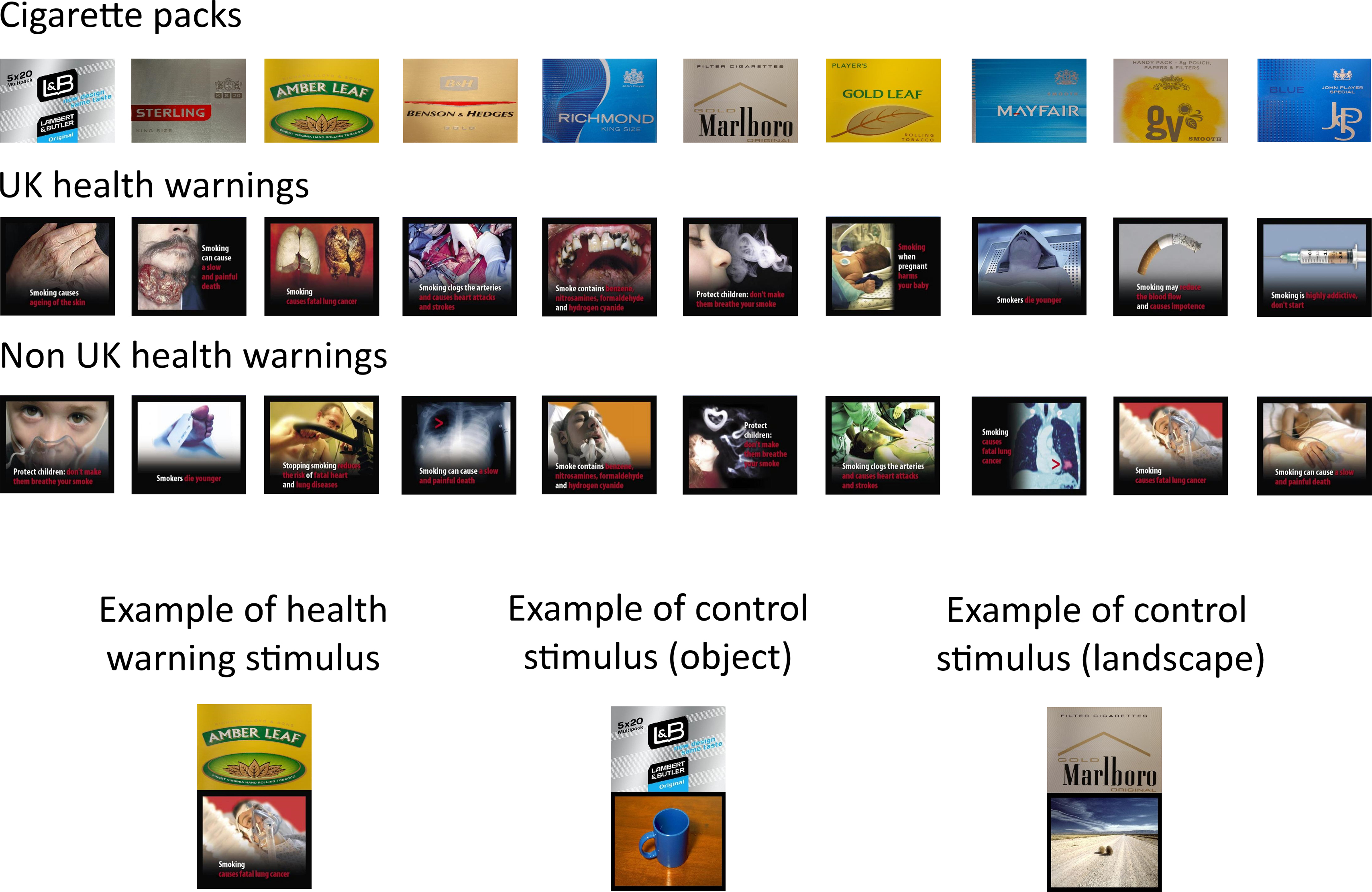

Supplement: Supplementary file 2 [file mmc2.jpg]
